# Understanding the Mechanisms through Which Family Risk Affects Adolescent Mental Health: A Model of Multisystemic Resilience in Context

**DOI:** 10.3390/children9040546

**Published:** 2022-04-12

**Authors:** Margherita Cameranesi, Linda Theron, Jan Höltge, Philip Jefferies, Michael Ungar

**Affiliations:** 1 Faculty of Health, Dalhousie University, Halifax, NS B3H 4R2, Canada; margherita.cameranesi@dal.ca (M.C.); j.hoeltge@protonmail.com (J.H.); philip.jefferies@dal.ca (P.J.); michael.ungar@dal.ca (M.U.); 2 Department of Educational Psychology, University of Pretoria, Pretoria 0027, South Africa; 3 Department of Psychology, University of Hawai’i at Manoa, Honolulu, HI 96822, USA

**Keywords:** conduct problems, externalizing mental health, family adversity, majority world, minority world, moderated mediation, multisystemic resilience, youth

## Abstract

There is substantial evidence that exposure to family adversity significantly and negatively impacts positive adolescent development by placing adolescents at increased risk of experiencing developmental difficulties, including conduct problems. Although the mechanisms responsible for these effects are still largely unknown, a novel line of inquiry in the resilience field conceptualizes positive adaptation, following exposure to atypical adversity, as resulting from complex interactions of systems at multiple ecological levels. The purpose of the present analysis was to apply this multisystemic resilience framework to the study of positive adaptation following exposure to family adversity in a sample of Canadian adolescents (*n* = 230; mean age 16.16, *SD* = 1.38) and South African adolescents (*n* = 421; mean age = 15.97, *SD* = 1.19) living in economically volatile communities dependent on the oil and gas industry. Cross-sectional survey data were used to investigate the mechanisms through which family adversity exercises its impact on adolescent conduct problems by accounting for their caregiving, peer, and community resources. Results of two moderated mediation analyses showed that family adversity impacts adolescent externalizing mental health negatively, via disrupted caregiving, when other resources are also considered. For the Canadian adolescents, these negative impacts were protectively moderated by peer support, but not moderated by appreciation for community traditions. In contrast, peer support showed no significant protective effect for the South African sample, while a strong appreciation for community traditions was positively and significantly associated with conduct difficulties. Contextual dynamics (e.g., social unrest) provide a plausible explanation for the discrepant results and bring attention to the importance of theorizing resilience in context.

## 1. Introduction

Externalizing mental health difficulties affect 5–10% of the world’s adolescent population [1,2]. Conduct disorder, which is a commonly reported externalizing difficulty (particularly among boys and younger adolescents) [3], is characterized by repeated violation of the basic rights of others (e.g., aggression to peers/animals) and/or major age-appropriate societal norms or rules [4,5]. It is ubiquitously associated with adolescent exposure to family adversity (i.e., one or more major stressful events within the immediate family context) [6,7]. Often, conduct difficulties preface poor educational outcomes and vulnerability to substance abuse, depression, and suicidality, as well as criminality and incarceration [7,8,9,10,11,12]. Such consequences have a high personal and societal cost.

Still, not all adolescents who are exposed to family adversity become conduct disordered. Put differently, some show resilience to the negative sequalae associated with family adversity. Resilience is defined as the capacity of a system (e.g., an adolescent) to maintain positive functioning (e.g., mental health or school engagement) despite exposure to a stressor that has the potential to undermine that system’s functioning [13,14]. Given the high personal and societal cost of conduct difficulties, it is imperative to understand the mechanisms that protect adolescents from developing conduct difficulties when they are challenged by family adversity [15]. This article responds to that imperative.

To do so, it draws on survey data generated by adolescents who participated in the Resilient Youth in Stressed Environments (RYSE) study. The RYSE study was specifically interested in the resilience of young people from communities dependent on an energy extraction industry, given this industry’s association with multiple risks at multiple system levels [16]. Typically, the energy extraction industry demands long hours from its workers, attracts migrant labour, is characterised by a boom-bust economy, and can pollute the immediate and adjacent physical ecologies [17]. In particular, these risks are believed to negatively impact young people’s family and community systems (e.g., by disrupting family relationships, increasing marital conflict, and increasing community conflict over scarce resources) [17,18].

RYSE was framed by a multisystemic understanding of resilience [16,19]. From this perspective, the capacity for positive functioning in the face of significant stress is informed by resources that are distributed across multiple systems and levels, with emphasis on resource-fit within the situational and cultural context [19,20]. Put differently, adolescent resilience requires personal (biological system; psychological system), relational (social system), and/or environmental (institutional system; built/natural ecological system) resources that are contextually congruent. To better understand the contextual congruence of resources that support positive functioning, RYSE purposefully included adolescents from a communities that are dependent on an energy extraction industry in the minority world (Canada (CA)) and majority world (South Africa (SA)). Majority world contexts are countries in which most of the world’s population lives; poverty and related resource constraints are pervasive, and enabling services/supports are largely unavailable or inaccessible [21,22]. As explained elsewhere [16], the CA and SA community choices potentiated exploration of the “heterogeneity in the factors and processes associated with resilience in both the Global South and the Global North” (p. 3).

As in much of the resilience literature, studies of the mechanisms that protect adolescents from developing conduct difficulties when they face family adversity foreground young people living in the minority world (e.g., North America, Europe, and Australia). It is unclear whether the mechanisms identified in these studies support adolescent resilience to the negative effects of family adversity in a majority world context. In juxtaposing the mechanisms through which family adversity impacts adolescent mental health in minority and majority world contexts, this article underscores the criticality of contextual factors to risk and resilience [13,19]. It encourages practitioner skepticism of a one-size-fits-all explanation of risk and resilience mechanisms. The resulting insights will be especially valuable to practitioners working with youth exposed to family adversity and typical majority world stressors (e.g., high social unrest), as well as those working with youth exposed to family adversity in contexts of lower social volatility.

### 1.1. Family Risk, Parenting, and Adolescent Conduct Difficulties

Family adversity can be defined as one or more significantly stressful events within a nuclear family unit, including the death of a parent/caregiver or sibling; severe/chronic parental/caregiver conflict and/or domestic violence; parental divorce; caregiver dysfunctionality (i.e., substance abuse, physical illness, or mental illness); parent/caregiver incarceration; and/or foster home placement [23]. Typically, cumulative family adversity has more pronounced negative effects. A plethora of studies has provided evidence of a dose-response relationship between family adversity and conduct disorders [24,25,26]. For instance, Bevilacqua et al. [7] investigated the associations between several family adversity events, (i.e., parental separation, depression, substance use, and intimate partner violence) and adolescent mental health, including behavioral difficulties, in a nationally representative cohort of over 8000 UK adolescents. The authors found a dose-response association between family adversity events and adolescent conduct problems in which a greater number of family adversities was associated with more severe conduct difficulties in study participants. Additionally, poor family caregiving characterized by harsh parenting and corporal punishment showed the strongest association with adolescent conduct problems.

Essentially, family adversity negatively affects adolescent mental health by jeopardizing adolescent access to quality caregiving [27,28,29]. For instance, maternal psychiatric symptoms can increase vulnerability in adolescents exposed to family adversity due to their effects on specific parenting behaviors, such as those relating to discipline or the expression of affection [30,31,32,33]. Specifically, mothers who experience trauma and severe mental health problems are more likely to perform inconsistent and harsh parenting, which is a significant predictor for adolescent conduct problems [7]. In addition, less engaged parents are unlikely to monitor their children’s peer associations, possibly allowing friendships with antisocial peers to flourish [34]. In contrast, supportive, quality caregiving matters for adolescent resilience [13]. In fact, the large cross-country network analysis conducted by Höltge et al. [35] showed that supportive caregiving was typically central to the multisystemic network of resources associated with positive adolescent outcomes across diverse contexts.

### 1.2. Peer Support and Adolescent Resilience to Conduct Difficulties

While adolescents with conduct difficulties may struggle to make friends and sustain friendships [36], supportive relationships with peers—especially prosocial ones—can discourage and/or mitigate conduct disorder [34], including in the context of family adversity [37,38]. For instance, Hopkins et al. [37] investigated the differential influence of several individual, family, community, and cultural resilience-enabling resources in predicting emotional and behavioral difficulties in a sample of 1021 Australian Aboriginal adolescents (12–17 years) exposed to different levels of family risk. The authors identified peer support to be uniquely associated with fewer behavioral difficulties for high family-risk youth with no benefits for youth in contexts of relatively low family risk. Essentially, supportive, prosocial peer relationships protect adolescents against the negative effects of family adversity by providing opportunities for adolescents to connect to prosocial role models (adult and peer); build mutually respectful relationships around shared interests; learn and practice prosocial behavior, emotional regulation, and effective problem solving (all of which are typically absent in families challenged by adversity); and access other resilience-enabling resources [37,39,40,41,42,43].

However, some studies dispute the protective effects of peer support when adolescents live in disadvantaged neighborhoods [44,45]. Typically, this relates to the quality of peer support. Specifically, neighborhood socioeconomic disadvantage is associated with poorer quality peer support (e.g., peers that endorse antisocial values or encourage delinquent/defiant behavior) and/or fewer opportunities to engage in constructive downtime activities with prosocial peers [46], which may have negative effects (albeit small to moderate) on adolescent conduct [47,48].

### 1.3. Appreciation for Community Traditions and Adolescent Resilience to Conduct Difficulties

Various resilience studies have reported that youth appreciation for their community’s traditions is associated with positive adjustment to adversity [13,49]. Community traditions typically have protective value because they facilitate organized activity that supports youth access to prosocial adults and prosocial peers; advances a sense of belonging; encourages a powerful racial/ethnic identify; and/or offers opportunities to learn about cultural heritage [13,50,51,52]. Similarly, organized community activity that engages youth in prosocial initiatives has the advantage of encouraging youth endorsement of prosocial values [50]. In addition, when community traditions encourage a sense of collective efficacy, young people are less vulnerable to negative or deviant socialization by peers with antisocial values [53]. Unfortunately, community disadvantage (e.g., widespread poverty) is associated with reduced collective efficacy and related decreased communal effort to informally control antisocial adolescent behavior in public neighborhood spaces [47,54]. Reduced collective efficacy invariably translates into adolescent vulnerability to negative or deviant socialization by peers with antisocial values.

Exposure to adversity may strengthen the association between young people’s identification with a collective (e.g., their community and its traditions) and conduct difficulties, particularly if that collective endorses antisocial behaviors [55]. For example, during adolescence, young people go through a process of identity transformation that leaves them vulnerable to identity confusion [56], and the distress stemming from this form of identity uncertainty has been associated with support of extremist views and actions [57]. As antithetical as it may seem, identifying with an antisocial or unconventional collective has nevertheless been associated with resilience among populations of marginalized and disenfranchised youth, including those whose challenges are compounded by family adversity [43,58].

### 1.4. Family Risk and Adolescent Resilience to Conduct Difficulties

#### 1.4.1. The Canadian Context

It is estimated that more than 60% of CA adolescents experience at least one adverse family event, including parental divorce or separation, exposure to intimate partner violence, parental death, and serious parent mental health illness [59], with one-third of CA adolescents estimated to experience two or more of these events. The literature points to the significant role that some factors play in promoting prosocial behavior in North American adolescents in the context of family adversity, including positive family functioning, peer support, and appreciation for community traditions [23,39,41,60]. Appreciation for community traditions is especially prominent in accounts of resilience among Indigenous Canadian youth [52], while its role as a resilience enabler for racial majority adolescents (White/Caucasian) is unclear. Positive aspects of caregiving (e.g., warmth, consistency, and availability) have been repeatedly linked to positive mental health in North American adolescents exposed to family adversity [29,61,62,63]. Supportive peer relationships and appreciation for community traditions have also been associated with positive adaptation in many stressful contexts, including family adversity (e.g., [64,65,66,67]). For example, in a CA study, Cameranesi et al. [41] investigated the resilience-enabling resources of 13 youth (ages 9–17) who had experienced exposure to intimate partner violence by conducting in-depth face-to-face semi-structured interviews. Using inductive thematic analysis and a constant comparative method, the authors identified adequate family caregiving and peer support among the most relevant resilience-enabling resources reported by these group of CA youth. Similarly, Rousseau et al. [60] investigated the associations between community connection, exposure to adversity, and sympathy for violent radicalization in CA college students by conducting a mixed-method study involving a large online survey of students at eight colleges. The study results showed the existence of complex associations between community connection and youth behavioral difficulties. Although the results suggested that, in youth exposed to adversity, a strong appreciation for community traditions can act as a protective anchorage, they also indicated that connection with violent radical groups may accentuate othering processes and legitimize violence toward the outgroup, thereby increasing youth conduct difficulties.

#### 1.4.2. The South African Context

As in many other majority world contexts, family adversity abounds in SA [68]. Typically, such adversity includes poverty, family violence, severe/chronic parental/caregiver conflict, parental/caregiver incarceration, parental/caregiver mental illness and/or substance abuse, child-caregiver separation, and/or orphanhood. Despite the high incidence of family risk in SA, very few studies have investigated what protects adolescents from challenged families from developing conduct difficulties [51]. Instead, studies have mostly investigated, and confirmed, that family adversity exposure (e.g., exposure to intimate partner violence) is significantly associated with conduct disorders (especially for boys) and other mental health problems among SA adolescents [69,70,71].

A notable exception is the study by Casale et al. [72] including 2477 adolescents, and their caregivers (96% isiZulu-speaking), from two resource constrained communities in KwaZulu Natal. More than a third of the adolescents were orphans; at least 40% reported hunger. Greater social support for caregivers, positive parenting practices, and better caregiver mental health were associated with less severe conduct difficulties. Similarly, a study of 616 adolescents (37.9% from single-parent or reconstituted family) in a low-income, violent community in Cape Town showed that maternal, paternal, and/or immediate family support attenuated conduct difficulties [73].

Interestingly, in the Humm et al. [73] study, peer support was significantly and positively associated with conduct disorders. While the association was weak and showed little practical significance, it fits with prior concerns about the intersectionality of neighborhood disadvantage, peer support quality, and conduct disorder [44,45,47,48]. Subsequent SA studies have reported similar non-protective effects of peer support on other adolescent mental health outcomes (e.g., depression) in disadvantaged, insurgent neighborhood contexts [71].

The SA resilience literature associates youth resilience with their engagement in positive family and community traditions that promote connectedness, a powerful personal and collective identity, and access to enabling cultural heritage [51]. While some SA adolescents believe that youth capacity for resilience is intertwined with community/collective capacity for lawfulness and prosocial accountability [74,75], many others endorse collective protest action. Collective protest—typically accompanied by unrest, violence, and destruction of property—is frequently understood as a legitimate response to the chronic and dehumanizing structural constraints that characterize many SA communities [76]. Notwithstanding the sociopolitical value of an insurgent collective identity, its potential to normalize violence and destruction is concerning. In SA communities where violence and gangsterism are the norm, young people report that hopefulness and prosocial behaviors are seldom endorsed by the collective [77]. In contrast, SA young people report positive developmental outcomes when their families and communities represent and encourage a prosocial collective identity [51,78].

### 1.5. The Present Study

**Figure 1:**
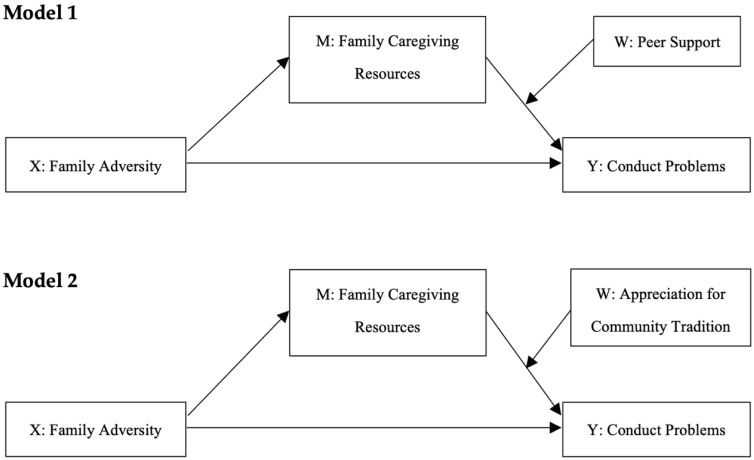
Conceptual models representing the two moderated mediation models tested in the study.

The objective of our investigation was to examine the mechanisms by which family adversity can negatively impact conduct problems in a sample of CA and SA adolescents, and the protective factors that promote their resilience to that impact. To this end, we tested two moderated mediation models involving the same mediation mechanism, but different moderators, to investigate the role that peer support and appreciation for community traditions play in buffering the negative effects of family adversity on conduct problems through family caregiving resources. Based on a multisystemic resilience-in-context framework [19], and current understandings of the mechanisms through which family adversity affects adolescent conduct difficulties [23,37], we propose a mediation model in which family adversity (X) leads to poor caregiving resources (M) that negatively impact adolescent adjustment, as shown by adolescents reporting conduct problems (Y). However, according to our model, when adolescents who experience family adversity have a supportive peer group or appreciate their community’s traditions, these act as buffering mechanisms that protect them from experiencing severe conduct difficulties. That is, in our model, the effect of family adversity on adolescent conduct problems through family caregiving is hypothesized to be contingent on the level of support adolescents receive from their peers and the strength of their appreciation for their community’s traditions, with a stronger positive effect of family adversity on conduct problems for adolescents with a less supportive peer group and less appreciation for their community’s traditions (see Figure 1 for a graphical representation of these effects).

Using conditional process analysis, two moderated mediation models were tested in a sample of CA and SA adolescents with the aim of exploring similarities and differences in the conditional nature of the mechanisms by which family adversity impacts conduct problems in these two groups. Conditional process analysis represents an approach to data analysis that integrates a mediation component with a moderation component into a single moderated mediation model [79]. The use of moderated mediation analysis made it possible to simultaneously investigate the direct and indirect pathways through which family adversity impacts adolescent conduct problems (the mediation component), and whether these pathways are dependent on a third variable (the moderation component). Additionally, the inclusion of adolescents from a minority world context (i.e., CA) and a majority world context (i.e., SA) made it possible to test for the existence of context-dependent moderating effects. Specifically, in both samples of adolescents, the potential role that family caregiving may play in mediating the association between family adversity and conduct problems was investigated, as well as whether peer support and adolescent appreciation for their community’s traditions moderate these indirect mechanisms. The following two sets of hypotheses informed these analyses.

**H1—Context-independent hypotheses.** Given the substantial evidence of the negative impact of family adversity on normative child development [6,7], it was hypothesized that family adversity directly affects adolescent mental health by increasing the severity of the conduct problems experienced by adolescent RYSE participants in CA and SA. That is, in both samples, it was expected that adolescents who are exposed to more family adversities would report more severe conduct difficulties, compared with adolescents who are exposed to a fewer number of family adversities, independently from adolescent family caregiving, peer support, and appreciation for community traditions. Additionally, given the literature linking family adversity to disrupted/poor caregiving [30,33], it was hypothesized that, in both samples, the direct effect of family adversity on adolescent conduct problems is mediated by family caregiving, so that, for both samples, exposure to more family adversities is associated with more severe conduct difficulties because of the poorer caregiving that these adolescents experience within their families (see Figure 1).

Further, it was hypothesized that, for both CA and SA adolescents, the indirect effect of family adversity on adolescent conduct problems through family caregiving is moderated by adolescent appreciation for community traditions. Specifically, it was anticipated that the indirect effect of family adversity on conduct problems through family caregiving is attenuated in adolescents who have greater appreciation for their community’s traditions, compared to their counterparts, who experience the same level of family adversity and family caregiving, but have less appreciation for their community’s traditions (see Figure 1 for details).

**H2—Context-dependent hypotheses.** A context-dependent hypothesis was formulated based on the literature suggesting that peer supports are regularly reported to have a protective effect in the North American context [41,80], while its value is questionable for adolescents living in disadvantaged neighborhoods, such as the RYSE SA community [71,73]. It was hypothesized that the indirect effect of family adversity on adolescent conduct problems through family caregiving is more likely to be moderated by peer support in the CA context than in the SA context. Thus, it was anticipated that, for CA adolescents, who have a more supportive peer group, the indirect effect of family adversity on conduct problems through family caregiving would be attenuated compared to their counterparts, who experience the same level of family adversity and family caregiving but have a less supportive peer group (see Figure 1). These effects were expected to be somewhat attenuated or null in the SA sample.

## 2. Materials and Methods

### 2.1. Procedure

The data used for the analysis described in this paper were collected during the RYSE project. RYSE, a 5-year (2017–2022) research study, investigated youth resilience in two communities heavily dependent on the energy extraction industry, and, therefore, susceptible to boom-and-bust economic cycles, family risk, and community risk: Drayton Valley, CA, and Secunda/eMbalenhle, SA [16]. Institutional Review Board (IREB) approval was obtained at the universities representing the affiliations of the two principal investigators in CA (Health Sciences Research Ethics Board, Dalhousie University, #2017-4321) and SA (Faculty of Health Sciences Research Ethics Committee, University of Pretoria, #UP17/05/01).

The moderated mediation analysis described in this paper used data extracted from a cross-sectional survey that was conducted in 2018 by interviewing two purposive samples of youth aged 13–24, one in the CA site (*N_ca_* = 500) and one in the SA site (*N_sa_* = 600). At the time of the survey, both communities were experiencing an economic downturn in the oil and gas industry and its associated challenges, involving, for example, job insecurity, reduced income, unemployment, family conflicts, and mental health problems. Additionally, as in most disadvantaged SA neighborhoods [76,81], the SA community was typified by structural disadvantage (e.g., inadequate housing and crowded living conditions) and social disorder (e.g., frequent violent protests in response to poor service delivery and local government corruption), with a growing sense that ‘protest culture’ characterizes this community’s culture [82]. Gangsterism, destruction of public property, and looting were common [83].

At both sites, a Local Advisory Committee (LAC), consisting of local youth and adults, was assembled to support the research team in planning and implementing all research activities, including participant recruitment. In collaboration with the LACs, a preliminary survey was developed and pilot tested with a small sample of respondents who were LAC members or had previously participated in RYSE qualitative work (*N_ca_* = 6; *N_sa_* = 6). The survey was then modified based on the respondents’ feedback (i.e., some items were added, while others were deleted). The final full survey contained a variety of items, including self-report measures assessing respondents’ multisystemic resilience-enabling resources and mental health, as well as questions assessing respondents’ sociodemographic characteristics (see next subsection for details).

Participants were recruited via social media and community-based advertising, classroom presentations, referrals, and snowball sampling (i.e., word of mouth). At both sites, the survey was administered to participants, either in small groups or individually, in a paper-pencil format in schools and community centers by trained local research assistants and members of the research team. To be included in the survey, participants had to be residents of the respective research communities, between 13 and 24 years of age, and proficient in English. To address literacy issues, the survey items were read aloud to study participants who requested it. Prior to participation, informed consent was obtained by all study participants and by parents/guardians of minors (adolescents younger than 18). CA adolescents received $25 CAD cash for their participation, while SA adolescents a ZAR150 (i.e., about $15 CAD) supermarket voucher. The incentive amounts were advised by the LACs.

### 2.2. Participants

As the overarching aim of this analysis was to investigate the mechanisms through which family adversity impacts adolescent mental health, the present study subsamples CA and SA adolescent (i.e., 13–18 years) survey respondents. Additionally, only respondents with complete data on all study variables were included in this investigation (*N_ca_* = 230; *N_sa_* = 421). A description of the two groups of adolescents is provided next.

#### 2.2.1. Canada

The CA sample included a total of 230 adolescents aged 13–18 years (mean age = 16.16, *SD* = 1.38). This group was almost evenly divided between biological sex, including 128 girls (55.7%) and 102 boys (44.3%). Most adolescents self-identified as being White (*n* = 184, 80.0%), while the remaining self-identified as being Indigenous (*n* = 27, 11.6%) and Black or of mixed race/ethnicity (*n* = 19, 8.4%). At the time of completing the survey, most CA adolescents were attending school (*n* = 215, 93.5%) and lived with both parents (*n* = 135, 58.7%) or only one parent (*n* = 65, 28.3%).

#### 2.2.2. South Africa

The SA sample included a total of 421 adolescents aged 14–18 years (mean age = 15.97, *SD* = 1.19). This group included slightly more girls (*n* = 266, 63.2%) than boys (*n* = 155, 36.8%). Most adolescents self-identified as being Black/African (*n* = 328, 77.9%), while the remaining self-identified as being White (*n* = 82, 19.5%) or of mixed race/ethnicity (*n* = 11, 2.6%). At the time of completing the survey, most SA adolescents were attending school (*n* = 412, 97.9%) and lived with both parents (*n* = 188, 44.7%) or only one parent (*n* = 116, 27.5%).

### 2.3. Measures

#### 2.3.1. Family Adversity

Family adversity was measured in both samples using a 9-item adaptation of the Family Adversity Scale [23]. Respondents were asked to report whether or not (0 = no, 1 = yes), at any time in the past, they had experienced nine family adversities, including living in a foster home, the death of a family member (caregiver or sibling), exposure to severe parental/caregiver conflict or intimate partner violence, parental divorce, and caregiver substance use problems, incarceration, or severe physical/mental health problems. These adverse events represent very common family risk factors that are customarily included in measures of family risk [84], as they have been consistently linked to increased conduct disorders in childhood and adolescence [7]. The original scale contains a 10th item asking respondents to indicate whether they had ever been separated from one or both parents. This item was excluded from the survey because it was anticipated that a large proportion of the study participants may not be living with a parent (e.g., due to parental divorce or traditional African kinship rearing practices). The items were summed to provide an indicator of the degree of family adversity experienced by respondents (scores = 0–9), with higher scores indicating higher family adversity. Reliability for the CA sample was *Ω* = 0.72 [0.62, 0.77], and for the SA sample, *Ω* = 0.53 [0.43, 0.61].

#### 2.3.2. Family Caregiving

Relevant items from the 28-item Child and Youth Resilience Measure (CYRM-28 [49]) were used to evaluate the quality of the caregiving adolescent respondents were experiencing at the time of completing the RYSE survey. Grounded in a multisystemic resilience framework [19], this measure includes 28 items that ask respondents to rate on a 5-point Likert scale (0 = not at all, 4 = a lot) their individual, relational, and contextual resources. Seven items ask participants to rate the quality of the physical and psychological caregiving they are receiving (e.g., “I feel safe when I am with my family”, “My family have usually supported me throughout life”, and “My family stands by me during difficult times”). Individual scores on these seven items were summed to generate a single total score providing a measure of respondents’ family caregiving, with higher sum scores indicating better caregiving. Reliability for the CA sample was *Ω* = 0.88 [0.85, 0.91], and for the SA sample, *Ω* = 0.79 [0.75, 0.82].

#### 2.3.3. Peer Support

Perceived peer support was assessed, asking both CA and SA adolescents to indicate on a 4-point Likert scale (0 = never true, 3 = always true) how much they felt supported by their friends, using the following four items derived from the 4-H Study of Positive Youth Development: “I trust my friends”, “I feel my friends are good friends”, “My friends care about me” and “My friends are there when I need them” [85]. Responses to the four items were summed to obtain an overall perceived peer support score, with higher scores indicating greater perceived peer support. Reliability for the CA sample was *Ω* = 0.91 [0.88, 0.93], and for the SA sample, *Ω* = 0.84 [0.80, 0.87].

#### 2.3.4. Appreciation for Community Traditions

A single-item measure of appreciation for community traditions was extracted from the CYRM-28 [49]. Adolescent participants reported on a 5-point Likert scale (0 = not at all, 4 = a lot) the degree to which they enjoyed their community traditions.

#### 2.3.5. Conduct Problems

To evaluate the conduct problems experienced by the CA and SA adolescents who completed the RYSE questionnaire, an adapted version of the Delinquency Scale [86] was included in the RYSE survey. The measure used in RYSE included 6 items that asked respondents to rate on a 5-point Likert scale (1 = never, 5 = 5 + times) how often they had performed a series of problem behaviours, including stealing something from a store, getting into trouble with the police, hitting or beating up someone, damaging property, carrying a weapon, and bullying someone. The single item scores were summed to obtain an overall conduct problem score, with higher scores indicating more severe conduct problems. Reliability for the CA sample was *Ω* = 0.81 [0.73, 0.86], and for the SA sample, *Ω* = 0.67 [0.60, 0.73].

#### 2.3.6. Sociodemographic Characteristics

The age (in years) and biological sex (1 = female, 2 = male) of CA and SA adolescents were assessed using a set of questions on respondents’ sociodemographic characteristics that were specifically developed for the purposes of the RYSE project.

### 2.4. Statistical Analysis

#### 2.4.1. Model Estimation

A series of robust moderated mediation analyses were conducted with ordinary least square path analysis using the PROCESS Macro v4.0 [79] for IBM SPSS v27 [87], to test our research hypotheses regarding the moderated mediation effects included in the conceptual model presented in Figure 1. A moderated mediation analysis is a special case of conditional process analysis involving a regression model that combines a mediation component with a moderation component to investigate the conditional nature of the mechanisms by which an antecedent variable X (i.e., predictor) transmits its effect on a consecutive variable Y (i.e., outcome) through a mediator M, and testing hypotheses about such conditional effects [88].

In both models tested, the predictor was the continuous variable family adversity, which ranged between 0 and 9 and represented the number of family adverse events the CA and SA adolescents experienced before completing the RYSE survey. In both moderated mediation models that were estimated, family adversity was used to predict adolescent conduct problems, which was a continuous variable representing the severity of the behavioral difficulties experienced by the study participants (range = 6–30). In the models, this direct effect was hypothesized to be mediated by a continuous variable, derived from the CRYM-28, indicating the quality of the family caregiving adolescent participants were receiving at the time of completing the RYSE survey (range = 0–28). Additionally, the variables perceived peer support (range = 0–12) and appreciation for community traditions (range = 0–4) were separately included in Model 1 and Model 2, respectively, as potential moderators of the indirect effect of family adversity on adolescent conduct problems through family caregiving. Hence, in the moderated mediation models estimated, it was tested whether family adversity significantly impacts adolescent conduct problems through caregiving, and whether this effect varies by the level of peer support (Model 1) and appreciation for community traditions (Model 2) that adolescents experience. In the models, we also tested whether family adversity exerts a direct (i.e., independent of family caregiving) and nonconditional (i.e., non-moderated) effect on adolescent conduct problems. The covariates age and biological sex were also included in the analysis.

#### 2.4.2. Model Inference

Analytically, the moderated mediation models depicted in Figure 1 were tested by simultaneously estimating two direct effects and one conditional indirect effect [79]. In this model, the *direct effect of X on Y* (i.e., family adversity on adolescent conduct problems) was neither hypothesized to be mediated or moderated, nor was it estimated as such. Similarly, we also estimated the *direct effects of X on M* by testing whether family adversity (X) exerts a direct (i.e., non-mediated) and nonconditional (i.e., non-moderated) effect on family caregiving (Y). However, what is of most interest in this model is *the conditional indirect effect of X on Y* (i.e., family adversity on conduct problems), which was calculated as the product of the direct effect of X on M and the conditional effect of M on Y, conditioned on peer support (W) in Model 1 and collective identity (W) in Model 2. PROCESS uses ordinarily least square path analysis to calculate these effects and provides a test of significance for both the direct effects and the conditional indirect effect.

To account for potential issues with sample size, outliers, normality, and homoscedasticity, a robust regression using bootstrapping (95% confidence intervals with 50,000 bootstrap samples) was applied [79,89]. When using bootstrapping, a bootstrap confidence interval (bCI) that does not include zero indicates that the estimated parameter (i.e., effect) is statistically significant. To make inferences about the significance of the moderated mediation (i.e., to test whether the mediation is moderated at the significance level α = 0.05) in the two conditional process models tested here, we used the index of moderated mediation as defined by Hayes [79]. Additionally, in both models, to probe the moderation of the indirect effect, we used 95% bCIs, which provide more accurate estimates than the Johnson-Neyman approach, as they do not make any normality assumption on the distribution of the conditional indirect effect of X on Y [79].

## 3. Results

### 3.1. Descriptive Analysis CA and SA Samples

Means, standard deviations, and bivariate correlations were calculated on all study variables. Additionally, a X^2^ test and independent samples t-test were performed to test for potential significant differences between the CA and SA sample on all study variables. As can be seen in Table 1, as expected, in both CA and SA samples, family adversity was significantly negatively correlated with family caregiving and peer support, as well as significantly positively correlated with adolescent conduct problems. Additionally, as expected, a significant negative correlation was identified between family caregiving and conduct problems for both CA and SA adolescents. In both samples, peer support and appreciation for community traditions were significantly positively correlated with family caregiving, but negatively correlated with conduct problems; this correlation was statistically significant only in the CA sample. No multicollinearity issues were identified, as indicated by correlation coefficients that did not exceed 0.52 and values of variance inflation factor (VIF) that did not exceed 1.7. A shown in Table 2, the only significant differences between the CA and SA samples pertained to the peer support, appreciation for community traditions, and conduct problems the two groups of adolescents were experiencing at the time of competing the survey, with the CA adolescents reporting significantly more peer support (t = 3.12, df = 445.102, *p* = 0.002), appreciation for their community’s traditions (t = 2.5, df = 512.949, *p* = 0.013), and conduct problems (t = 2.82, df = 346.458, *p* = 0.005) than their SA counterparts.

### 3.2. Model 1

#### 3.2.1. Canada

**Figure 2:**
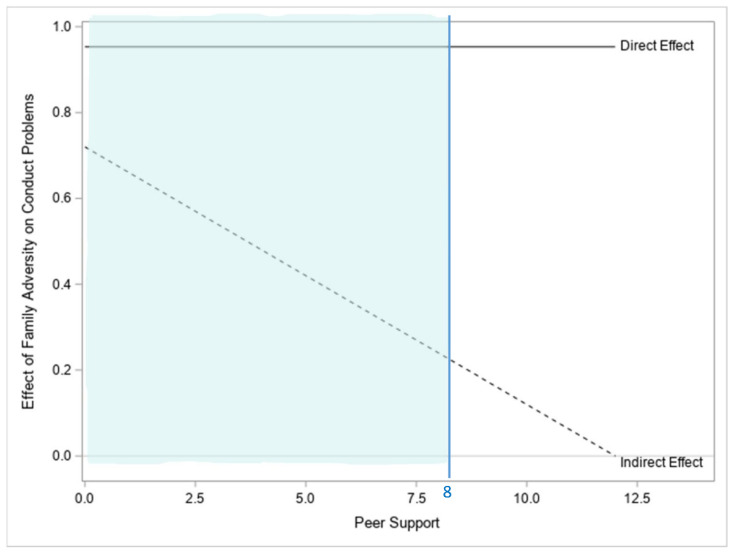
Visual representation of the conditional indirect and the direct effect of family adversity on the conduct problems of CA adolescents, with the indirect effect operating through family caregiving. The blue region to the left of the blue line represents the levels of peer support at which the indirect effect is statistically significant as indicated by bCIs.

The robust full moderated mediation model in which the effect of family adversity on adolescent conduct problems through family caregiving was modeled to be conditional on peer support explained 32.6% of the variance in conduct problems for CA adolescents (F(6223) = 10.899, *p* < 0.001). In this model, the index of moderated mediation is significantly different from zero, at the significance level α = 0.05, indicating that the mediation tested in the model is indeed moderated, or that the indirect effect of family adversity on adolescent conduct problems through family caregiving is dependent on the level of support the adolescents receive from their peers (coefficient b = −0.065, b*SE* = 0.026, 95% bCI = [−0.117, −0.015]). As can be seen in Table 3, family adversity has a significant negative impact on family caregiving. so that adolescents who experience a greater number of family adversities tend to report less positive family caregiving (b = −1.136, b*SE* = 0.206, 95% bCI = [−1.540, −0.726]). Additionally, as hypothesized, the effect of family caregiving on conduct problems is contingent on peer support, as evidenced by the statistically significant interaction between M and W in the model of Y (b = 0.057, b*SE* = 0.018, 95% bCI = [0.016, 0.087]). In this model, the conditional indirect effect of family adversity on adolescent conduct problems, through family caregiving (conditioned on peer support), is positive for low to moderate values of peer support and negative for high values of peer support, and significant only for low values of peer support (i.e., below 8), as indicated by the 95% bCI of the indirect effect when peer support is equal to 8 (b = 0.162, b*SE* = 0.076, 95% bCI = [0.025, 0.324]). That is, there is no significant effect of family adversity on conduct problems through family caregiving for moderate and high levels of peer support, while there is a significant positive effect at low levels of peer support below the value of 8, as indicated by the significant region identified using bCIs showed in Figure 2. Thus, in line with the study hypotheses, family adversity significantly increases the likelihood that CA adolescents will experience conduct problems by disrupting the caregiving they receive within their family and this effect is attenuated for adolescents with supportive peers. The direct effect of family adversity on externalizing difficulties quantifies how much two adolescents who differ by one adverse family event are estimated to differ in conduct problems, by holding constant family caregiving and peer support. In this model, as can be seen in Table 3, this direct effect is positive and significant (b = 0.953, b*SE* = 0.196, 95% bCI = [0.565, 1.330]). Therefore, two CA adolescents who differ by one adverse family event, but experience the same family caregiving and peer support, are estimated to differ by 0.919 units in conduct problems, with the adolescent experiencing more family adversity estimated to present significantly more externalizing problems. Hence, in line with the study hypotheses, family adversity significantly increases the likelihood that CA adolescents will experience conduct problems, independently from the support they receive from their family and peers.

Figure 2 displays a visual representation of the conditional indirect and the direct effect of family adversity on adolescent conduct problems, with the indirect effect operating through family caregiving. Additionally, this figure shows the levels of peer support at which this indirect effect is statistically significant by including the region of significance generated using bCIs (i.e., the blue region to the left of the blue line or below values of peer support equal to 8).

#### 3.2.2. South Africa

The robust full moderated mediation model in which the effect of family adversity on adolescent conduct problems through family caregiving was modeled to be conditional on peer support explained 22.4% of the variance in conduct problems for SA adolescents (F(6414) = 13.893, *p* < 0.001). For the SA sample, this moderated mediation model was not significant, as indicated by an index of moderated mediation that was not significantly different from zero at the significance level α = 0.05 (coefficient *b* = 0.000, b*SE* = 0.004, 95% bCI = (−0.008, 0.009)). Additionally, as can be seen in Table 3, the interaction term in this model is not significant (*b* = −0.002, b*SE* = 0.009, 95% bCI = (−0.020, 0.015)), indicating that, for the SA adolescents, the effect of family adversity on conduct problems through family caregiving resources is not contingent on peer support. The only two significant effects in this model are the direct effect of family adversity on family caregiving (*b* = −0.454, b*SE* = 0.146, 95% bCI = (−0.743, −0.172)), and conduct problems (*b* = 0.429, b*SE* = 0.099, 95% bCI = (0.236, 0.628)). In line with the study hypotheses, these effects indicate that, independent of peer support, family adversity significantly negatively impacts caregiving s, as well as the behavior of SA adolescents.

### 3.3. Model 2

#### 3.3.1. Canada

The robust full moderated mediation model, in which the effect of family adversity on adolescent conduct problems through family caregiving was modeled to be conditional on appreciation for community traditions, explained 28.3% of the variance in conduct problems for CA adolescents (F(6223) = 13.893, *p* < 0.001). Contrary to the study hypotheses, for the CA sample, this moderated mediation model was not significant, as indicated by an index of moderated mediation that was not significantly different from zero at the significance level α = 0.05 (coefficient *b* = −0.072, b*SE* = 0.067, 95% bCI = [−0.200, 0.065]). Additionally, as can be seen in the first portion of Table 4, the interaction term in this model is not significant (*b* = 0.064, b*SE* = 0.060, 95% bCI = (−0.054, 0.182)), indicating that, for the CA adolescents, the effect of family adversity on conduct problems through family caregiving resources is not contingent on their appreciation for community traditions. The only two significant effects in this model are the direct effects of family adversity on family caregiving (*b* = −1.136, b*SE* = 0.214, 95% bCI = [−1.557, −0.714]) and conduct problems (*b* = 0.943, b*SE* = 0.220, 95% bCI = [0.509, 1.378]). As hypothesized, these effects indicate that, independent of appreciation for community traditions, family adversity significantly negatively impacts caregiving, as well as the behavior of CA adolescents.

#### 3.3.2. South Africa

The robust full moderated mediation model, in which the effect of family adversity on adolescent conduct problems through family caregiving was modeled to be conditional on appreciation for community traditions, explained 21.7% of the variance in conduct problems for SA adolescents (F(6414) = 13.494, *p* < 0.001). In this model, the index of moderated mediation is significantly different from zero at the significance level α = 0.05, indicating that the mediation tested in the model is indeed moderated, or that the indirect effect of family adversity on adolescent conduct problems through family caregiving is dependent on the strength of adolescents’ appreciation for their community’s traditions (coefficient b = 0.028, b*SE* = 0.013, 95% bCI = (0.008, 0.057)).

As can be seen in Table 4, the first equation of Model 2 reflects what was found in Model 1: family adversity has a significant negative impact on family caregiving, so that SA adolescents who experience a greater number of family adversities tend to report less positive family caregiving (b = −0.454, b*SE* = 0.148, 95% bCI = (−0.744, −0.163)). Additionally, as hypothesized, the effect of family caregiving on conduct problems is contingent on appreciation for community traditions, as evidenced by the statistically significant interaction between M and W in the model of Y (b = −0.063, b*SE* = 0.019, 95% bCI = (−0.101, −0.024)).

**Figure 3:**
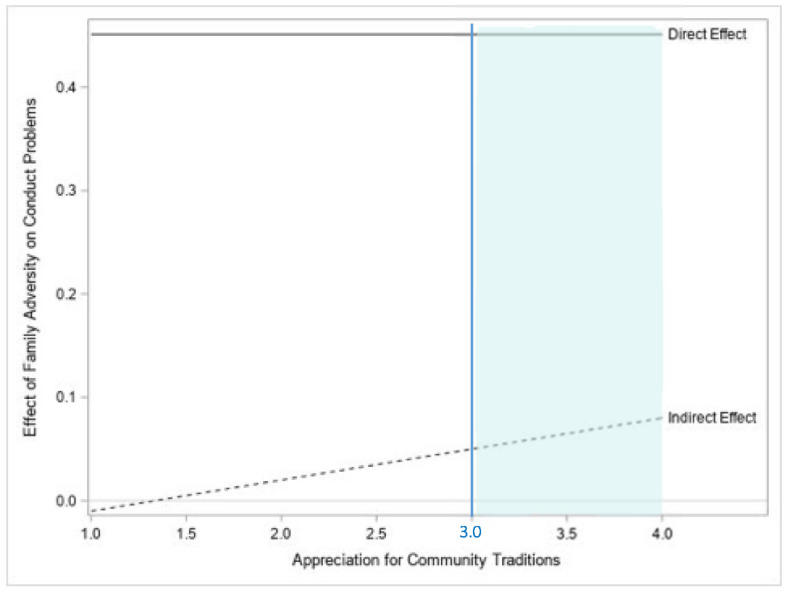
Visual representation of the conditional indirect and the direct effect of family adversity on the conduct problems of SA adolescents, with the indirect effect operating through family caregiving. The blue region to the right of the blue line represents the levels of appreciation for community traditions at which the indirect effect is statistically significant as indicated by bCIs.

Contrary to what was hypothesized, in this model, the conditional indirect effect of family adversity on adolescent conduct problems through family caregiving (conditioned on appreciation for community traditions) is negative for low values of appreciation for community traditions, and positive for moderate and high values of appreciation for community traditions, as well as being significant for high values of collective identity (i.e., at and above 3), as indicated by the 95% bCI of the indirect effect when collective identity is equal to 3 (b = 0.048, b*SE* = 0.024, 95% bCI = (0.009, 0.104)) and 4 (b = 0.076, b*SE* = 0.035, 95% bCI = (0.020, 0.157)). That is, there is no significant effect of family adversity on conduct problems through family caregiving for low levels of appreciation for community traditions, while there is a significant positive effect at moderate and high levels of appreciation for community traditions at and above the value of 3, as indicated by the significant region identified using bCIs showed in Figure 3. Thus, in line with the study hypotheses, family adversity significantly increases the likelihood that SA adolescents will experience conduct problems by disrupting the caregiving they receive within their family; however, contrary to what it was expected, this effect is amplified for SA adolescents with a greater appreciation for their community’s traditions.

Similar to what was found in Model 1, in this model the direct effect of family adversity on externalizing difficulties is positive and significant (b = 0.451, b*SE* = 0.105, 95% bCI = [0.244, 0.658]). Therefore, two SA adolescents who differ by one adverse family event, but have the same family caregiving and appreciation for community traditions, are estimated to differ by 0.451 units in conduct problems, with the adolescent experiencing more family adversity estimated to present significantly more behavioral problems. Hence, in line with the study hypotheses, family adversity significantly increases the likelihood that SA adolescents will experience conduct difficulties, independently from the support they receive from their family and their appreciation for community traditions.

Figure 3 displays a visual representation of the conditional indirect and the direct effect of family adversity on adolescent conduct problems, with the indirect effect operating through family caregiving. Additionally, this figure shows the levels of appreciation for community traditions at which this indirect effect is statistically significant, by including the region of significance generated using bCIs (i.e., the blue region to the right of the blue line, or at and above values of collective identity equal to 3).

## 4. Discussion

To better understand how family adversity impacts adolescent mental health in a majority and minority world context, two moderated mediation models were tested. The models were applied to survey data generated by adolescent RYSE participants who were purposively sampled from two oil-and-gas industry-dependent communities in CA and SA experiencing economic downturn. The SA community was additionally challenged by regular protests and related violence, as well as gangsterism [83]. Although the nature of the sample limits generalizability (especially to clinical adolescent populations), the results redress the relative inattention to the mental health resilience of majority world adolescents, and direct attention to how contextual dynamics play into risk and resilience.

Three hypotheses (two context independent; one context dependent) informed the moderated mediation analyses. The first context independent hypothesis theorized that being exposed to fewer family adverse events would protect both CA and SA adolescents against conduct problems, because they had access to quality caregiving. Indeed, the results of Model 1 for CA and Model 2 for SA showed that greater exposure to family adversity significantly increased adolescent risk of reporting conduct problems, by significantly decreasing the likelihood of adolescents reporting caregiving resources that promote and protect positive developmental outcomes. These results reinforce the criticality of caregiving resources to the mental health resilience of adolescents in the majority and minority world [15,35,90], including when these adolescents are exposed to family adversity. They also direct attention to the importance of protecting the indirect pathways of adolescent resilience. Put differently, they are a reminder that protecting adolescent mental health will require protecting the health and wellbeing of their caregivers [91]. The SA study by Casale et al. [72] is a case in point: it showed a significant association between caregiver health, caregiver access to social support, and lower levels of adolescent conduct difficulties.

The second hypothesis, which was also context independent, anticipated that the indirect effect of family adversity on adolescent conduct problems through family caregiving would be moderated by appreciation for community traditions. Specifically, it was anticipated that for CA and SA RYSE participants with a stronger appreciation for community traditions, the indirect effect of family adversity on conduct problems through family caregiving would be attenuated. Certainly, pre-existing resilience studies had reported positive effects when adolescents appreciate their community’s traditions [13,49,50,51,52,60], albeit not exclusively in the context of family adversity. A strong appreciation for community traditions did not buffer the negative effect of family adversity on CA adolescents’ conduct problems through family caregiving. This lack of buffering effect for CA RYSE participants, who mostly self-identified as White, fits with earlier reports of cultural factors (e.g., appreciation of community tradition) being poorly associated with the resilience of visible majority youth in CA [52,92]. In the SA sample, however, a strong appreciation for community traditions increased the indirect effect of family adversity on adolescent conduct problems through family caregiving. In other words, compared to SA RYSE participants who reported less appreciation for community traditions, having a stronger appreciation for community traditions significantly increased the conduct disorder risk of SA adolescents exposed to family adversity. This is, perhaps, not a surprising finding, given the chronic structural constraints that overwhelm the SA RYSE site and the recurring collective response involving violent protest and related lawlessness [83]. Indeed, the SA RYSE site’s culture has been described as ‘protest culture’ [82]. Moreover, protest is a recurring response across similarly constrained communities in SA [76]. In the context of enduring structural violence, a community that champions resistance and repeatedly embraces attitudes and behaviors that violate mainstream societal norms is potentially more powerful than one that tolerates continued marginalization and inequity [43,76]. Still, a strong appreciation for community traditions that endorse insurgent behaviors is unlikely to attenuate conduct difficulties. Mental health advocates who work in similarly angry and disenfranchised communities need to be cautious about promoting adolescent engagement in community traditions as a way of coping with stresses in the family context. Further, this unexpected positive effect of a strong appreciation for community traditions on conduct problems among the SA RYSE participants should be interpreted as a reminder of the social and structural determinants of mental illness, and the imperative of redressing those determinants [93]. Overall, the results suggest that the potential for community traditions to ameliorate conduct disorders in the face of family adversity should be viewed as relative to community dynamics and/or racial/ethnic identity.

The third hypothesis, which was context dependent, theorized that the protective value of peer support to adolescent conduct difficulties in the face of family adversity was more likely to be realized for the CA sample than the SA one. Our skeptical regard for the value of peer support to the SA sample’s mental health resilience related to peer support having been positively and significantly associated with adolescent conduct problems and other mental health difficulties when adolescents lived in a disordered or violent SA neighborhood [71,73]. The results, which showed that high peer support protected only the CA sample from the negative indirect impact that family adversity has on their behavior through family caregiving, substantiated this context-dependent hypothesis. Given how neighborhood dynamics play into the protective value of peer support [44,45,46,47,48,54], it is plausible that the absence of significant protective effects for the SA sample was an artefact of the social unrest and disorder that characterized the SA RYSE site [16,83]. While peer support did not influence the impact of family adversity on SA RYSE participants’ conduct disorders (as reported in Humm’s study [73]), its lack of significant protective effect for the SA sample cautions against one-size-fits-all understandings of what informs adolescent resilience [19,20]. Instead, it points to the salience of situational context; to which resources matter for adolescent mental health resilience to family adversity.

## 5. Limitations

It is important to acknowledge the hypotheses’ relatively narrow focus on caregiving, peer support resources, and appreciation for community traditions, as well as related limitations in understanding how biological, psychological, and ecological systems play into adolescent mental health resilience to family adversity [19]. In addition, while researchers are encouraged not to consider 0.70 as the gold standard for reliability [94], the reliabilities of the family adversity and conduct problems measures were low for SA. Additionally, the RYSE participants were recruited through purposeful sampling, rather than random sampling, and, therefore, they represent a subgroup of adolescents that may not be representative of the general adolescent population in majority and minority world contexts. Further, the data used for this analysis were cross-sectional; therefore, the order of antecedent and consequent variables tested in the estimated models could be questioned.

We used a single item to measure appreciation for community traditions. While a growing body of literature advocates for the acceptability of single-item measures [95,96,97], it is possible that a multi-item measure of young people’s engagement with/appreciation for community traditions would have prompted different insights. Additionally, the survey methodology did not allow insight into what the community traditions were or whether they fomented behaviours associated with conduct disorders.

The moderated mediation effects identified in this analysis should be replicated using longitudinal research designs that recruit large population-based samples of adolescents from majority and minority world contexts, and more comprehensive assessments of family adversity that include, for example, its frequency and impact on adolescent mental health. Additional covariates could also be entered into these analyses. Such future studies should also assess the quality of peer support (i.e., prosocial vs. antisocial peers) and use multiple-item measures to assess other resilience-enablers and their value in context. Ideally, follow-up studies should use mixed methods to better understand resilience-enablers in a given context at a specific point in time.

## 6. Conclusions

Notwithstanding the limitations, the research hypotheses informing this article can be used to design formal and informal interventions. Taken together, the results refute mono-systemic (e.g., adolescent or family focused) and contextually neutral explanations of adolescent mental health resilience when adolescents have experienced family adversity. Specifically, there is a need to add resources at multiple systemic levels; for example, by targeting the quality of peer supports, caregiving, and the potential for a young person to feel engaged with their community and appreciate their community’s traditions. In general, our findings echo previous research, in that there is value to considering the impact of each of these dimensions of an adolescent’s life, and that each can significantly ameliorate the effects of family adversity on mental health, provided these resources have contextual protective value [19]. Herein lies the challenge. There is a need to consider the differential impact [98] of various types of resources in a young person’s life and whether these resources are relevant. Prevention and intervention programs, targeting adolescent conduct problems, will be most successful if they are context specific and simultaneously address multiple systemic influences at the level of the individual, family, and community. What an adolescent in CA needs to overcome a difficult past will look quite different than a young person in SA, where the community risk factors reflect different social conditions. There, are however, also similarities across countries. Informed by our findings, we suggest that in both majority and minority world contexts adolescent mental health resilience to family adversity can be facilitated by increasing caregiver access to social support [72,91]. In minority world contexts, though, such as CA, encouraging better peer relationships and closer contact with an adolescent’s community, through initiatives such as mentoring programs or opportunities for a young person to contribute meaningfully through volunteer or paid activities, may be beneficial. Such benefits, though, are unlikely to be realized for youth in a country such as SA. Therefore, our research provides a cautionary note for program developers. In contexts where there is social injustice, and peer relationships are likely to lead to resistance to social norms, or where community involvement may manifest as participation in social unrest, those intervening to help young people will need to consider how a protective factor functions, and what resilience-promoting behaviour looks like. Where we see conduct disorder in a more orderly society, such as CA, that same pattern of conduct disorder may be associated with a search by young people to exercise their human rights or seek the means to meet their basic needs in a country such as SA, where public institutions are struggling to meet people’s needs. By thinking of resilience multisystemically, there is greater likelihood of identifying the best protective factors that best fit a specific context [43].

## Figures and Tables

**Table 1:** Intercorrelations among study variables disaggregated by country.

Variable	1	2	3	4	5	6	7
1. Sex	-	0.079	−0.082	0.071	−0.045	0.052	0.154 **
2. Age	0.166 **	-	0.144 **	−0.032	−0.075	−0.006	0.151 **
3. Family adversity	−0.059	0.140 **	-	−0.242 **	−0.122 *	−0.188 **	0.307 **
4. Family Caregiving	−0.004	−0.004	−0.151 **	-	0.283 **	0.524 **	−0.206 **
5. Peer support	−0.072	−0.104 *	−0.127 **	0.223 **	-	0.282 **	−0.081
6. Appreciation for Community Traditions	0.090 *	−0.023	0.016	0.321 **	0.204 **	-	−0.201 **
7. Conduct problems	0.313 **	0.054	0.206 **	−0.098 *	−0.217 **	−0.008	-

Note. Correlations above the diagonal relate to the CA sample (*n* = 230). Correlations below the diagonal relate to the SA sample (*n* = 421). No missing data. * *p* < 0.05, ** *p* < 0.01.

**Table 2:** Results of independent *t*-test examining significant differences between the CA and SA samples.

Variable	Canada		South Africa		*t*	*p*	Cohen’s *d*
	*M*	*SD*	*M*	*SD*			
Age	16.16	1.38	15.97	1.19	1.81	0.070	0.316
Family adversity	2.17	2.05	1.88	1.60	1.88	0.061	0.327
Family Caregiving	21.86	5.86	22.71	4.67	−1.91	0.057	−0.006
Peer support	8.88	2.89	8.16	2.70	3.12	0.002 *	0.422
Appreciation for Community Traditions	2.47	1.21	2.21	1.34	2.5	0.013 *	0.199
Conduct problems	9.43	4.75	8.44	3.22	2.82	0.005 *	0.419

Note. CA sample (*n* = 230). SA sample (*n* = 421). No missing data. * *p* < 0.01.

**Table 3:** Model coefficients for Model 1 in Figure 1.

			Canada			
	Family Caregiving (M)			Conduct problem (Y)		
	Coeff *b*	b*SE (b)*	95% bCI *(b)*	Coeff *b*	b*SE (b)*	95% bCI (*b*)
Constant	22.788	4.430	[14.067, 31.522] *	17.672	5.253	[5.583, 26.401] *
Family adversity (X)	−1.136	0.206	[−1.540, −0.726] *	0.953	0.196	[0.565, 1.330] *
Family caregiving (M)	-	-		−0.603	0.163	[−0.878, −0.232] *
Peer support (W)	-	-	-	−1.260	0.435	[−1.960, −0.269] *
M x W (interaction)	-	-	-	0.057	0.018	[0.016, 0.087] *
Age (covariate 1)	0.040	0.268	[−0.489, 0.562]	0.035	0.210	[−0.363, 0.452]
Sex (covariate 2)	0.612	0.712	[−0.824, 1.985]	1.380	0.518	[0.396, 2.410] *
	R^2^ = 0.164 *F*(3226) = 10.708, *p* < 0.001			R^2^ = 0.326 *F*(6223) = 10.899, *p* < 0.001		
			**South Africa**			
	Family Caregiving (M)			Conduct problem (Y)		
	Coeff *b*	b*SE (b)*	95% bCI *(b)*	Coeff *b*	b*SE (b)*	95% bCI (*b*)
Constant	22.479	3.125	[16.375, 28.692] *	8.338	2.830	[2.851, 13.891] *
Family adversity (X)	−0.454	0.146	[−0.743, −0.172] *	0.429	0.099	[0.236, 0.628] *
Family caregiving (M)	-	-	-	−0.006	0.077	[−0.156, 0.149]
Peer support (W)	-	-	-	−0.152	0.217	[−0.566, 0.293]
M x W (interaction)	-	-	-	−0.002	0.009	[−0.020, 0.015]
Age (covariate 1)	0.079	0.198	[−0.314, 0.458]	−0.157	0.119	[−0.223, 0.279]
Sex (covariate 2)	−0.134	0.469	[−1.056, 0.782]	2.579	0.337	[1.924, 3.253] *
	R^2^ = 0.023 *F*(3417) = 3.212, *p* < 0.05			R^2^ = 0.224 *F*(6414) = 13.892, *p* < 0.001		

Note. CA sample (*n* = 230). SA sample (*n* = 421). No missing data. * Significant bCI.

**Table 4:** Model coefficients for Model 2 in Figure 1.

			Canada			
	Family Caregiving (M)			Conduct problem (Y)		
	Coeff *b*	b*SE (b)*	95% bCI *(b)*	Coeff *b*	b*SE (b)*	95% bCI (*b*)
Constant	22.788	4.525	[13.871, 31.705] *	10.419	5.352	[−0.129, 20.966]
Family adversity (X)	−1.136	0.214	[−1.557, −0.714] *	0.943	0.220	[0.509, 1.378] *
Family caregiving (M)	-	-	-	−0.265	0.166	[−0.593, 0.063]
AfCT (W)	-	-	-	−1.589	1.407	[−4.361, 1.184]
M x W (interaction)	-	-	-	0.064	0.060	[−0.054, 0.182]
Age (covariate 1)	0.040	0.274	[−0.500, 0.581]	0.037	0.227	[−0.410, 0.485]
Sex (covariate 2)	0.612	0.717	[−0.800, 2.024]	1.657	0.510	[0.652, 2.661] *
	R^2^ = 0.164 *F*(3226) = 10.708, *p* < 0.001			R^2^ = 0.283 *F*(6223) = 8.637, *p* < 0.001		
			**South Africa**			
	Family Caregiving (M)			Conduct problem (Y)		
	Coeff *b*	b*SE (b)*	95% bCI *(b)*	Coeff *b*	b*SE (b)*	95% bCI (*b*)
Constant	22.479	3.142	[16.303, 28.655] *	4.178	2.061	[0.127, 8.230] *
Family adversity (X)	−0.454	0.148	[−0.744, −0.163] *	0.451	0.105	[0.244, 0.658] *
Family caregiving (M)	-	-	-	0.082	0.043	[−0.002, 0.167]
AfCT (W)	-	-	-	1.320	0.454	[0.426, 2.213] *
M x W (interaction)	-	-	-	−0.063	0.019	[−0.101, −0.024] *
Age (covariate 1)	0.079	0.198	[−0.314, 0.458]	−0.111	0.119	[−0.345, 0.123]
Sex (covariate 2)	−0.134	0.469	[−1.056, 0.782]	2.682	0.346	[2.001, 3.363] *
	R^2^ = 0.023 *F*(3417) = 3.212, *p* = 0.02			R^2^ = 0.217 *F*(6414) = 13.494, *p* < 0.001		

Note. CA sample (*n* = 230). SA sample (*n* = 421). No missing data. AfCT = Appreciation for Community Traditions. * Significant bCI.

## Data Availability

Qualified researchers can obtain the data from the corresponding author (linda.theron@up.ac.za). The data are not publicly available due to privacy concerns imposed by the IRBs that approved the study.
